# Highly stable and conformal Ti–organic thin films from sustainable precursors *via* atomic/molecular layer deposition towards green energy applications†

**DOI:** 10.1039/d4cc05425c

**Published:** 2024-12-06

**Authors:** Anish Philip, Umaid Lone, Olga Partanen, Eero Haimi, Maarit Karppinen

**Affiliations:** a School of Chemical Engineering, Aalto University FI-00076 Espoo Finland maarit.karppinen@aalto.fi

## Abstract

Thin-film deposition using sustainable precursors is required for various next-generation green energy applications. Here we report two atomic/molecular layer deposition processes for appreciably stable and conformal Ti–organic thin films and TiO_2_:organic superlattices with potential in *e.g.* battery, photocatalysis and thermoelectric applications. These processes are based on the safe and sustainable titanium isopropoxide as the titanium precursor.

## Abstract 2

Stable and conformal Ti–organic thin films from titanium isopropoxide and organic precursors (hydroquinone or benzene dicarboxylic acid) *via* ALD/MLD technique is reported; conformality is verified with lateral high-aspect-ratio structures.

## Graphical abstract

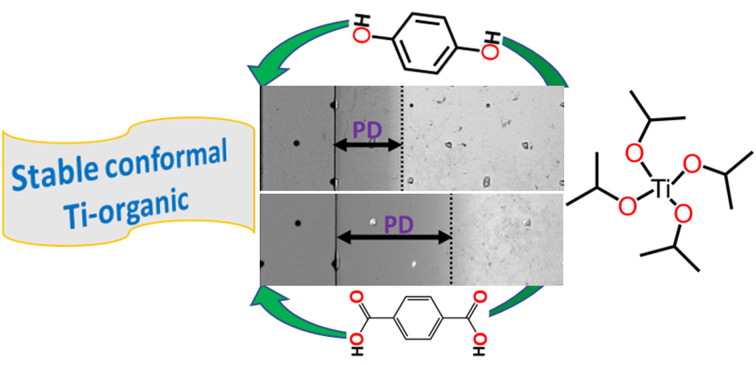

Hybrid metal–organic thin films fabricated through the currently strongly emerging atomic/molecular layer deposition (ALD/MLD) technique have attracted increasing interest as enablers of various next-generation applications, including green energy applications.^1–3^ For such functional hybrid thin films, titanium as an abundant, non-poisonous and safe element is an ideal metal component; once combined with a proper organic component, the resultant Ti–organic thin films have been already highlighted as attractive protective coatings for battery materials, especially in extending the lifetime of Li-ion batteries by preventing the direct interaction between the electrolyte and electrode materials.^4–6^ Metal–organic coatings are believed to be superior over their inorganic counterparts for the electrode protection, thanks to their better mechanical flexibility allowing better adjustment to the electrode volume changes during the battery charging/discharging.^6–9^ Flexible/wearable thermoelectrics is another example of potential application areas for the hybrid ALD/MLD thin films. Here especially useful would be different superlattice (SL) structures in which thin organic layers are embedded within the inorganic matrix, to provide enhanced mechanical flexibility and phonon-scattering interfaces to reduce the thermal conductivity, thereby enhancing the thermoelectric heat-to-electricity conversion efficiency.^10,11^

The ALD/MLD processes so far developed for Ti–organic thin films are mostly based on TiCl_4_ as the titanium precursor (Table 1). The major drawback related to the use of this precursor is the formation of hazardous/corrosive biproducts (HCl) that are harmful for the reactor parts and also for applications involving sensitive substrates. Another issue is the chlorine contamination in the targeted Ti–organic thin films which has been found to play a major role in the commonly observed instability of these films.^12,13^ Development of alternative chlorine-free ALD/MLD processes for Ti–organic thin films is thus essential for the overall process sustainability as well as film purity/stability.

**Table 1:** Previously reported optimized ALD/MLD processes for Ti–organic thin films

Titanium precursor	Organic precursor	*T*_dep_ (°C)	GPC (Å per cycle)	Ref.
TiCl_4_	Fumaric acid	200	0.9	14
	Ethylene glycol	90–115	4.5	15
	Glycerol	130	2.8	15
	2,4-Hexadiyne-1,6-diol	100	6	16
	4,4-Oxydianiline	160	0.3	17
	8-Hydroxyquinoline	85–150	6.5–1	18
	4-Aminophenol	120–160	10–11	19, 20
	Triethanolamine	150–195	5–2	21
	Hydroquinone	170	4.3	19
	*p*-Phenylenediamine	300	1.2	19

Ti(O^i^Pr)_4_	Glycine	225	1.1	22
	l-Aspartic acid	250	0.6	22
	Succinic acid	180	2.2	22
	Curcumin	300	3.9	23

TDMAT	Glycine	80–160	0.9–0.2	5
	Oxalic acid	100	2.7	4
	Succinic acid	100	0.9	4
	Glutaric acid	100	0.7	4
	3,6-Dioxaoctanedioic acid	100	0.6	4

In previous studies, halogen-free tetrakis(dimethylamino) titanium (TDMAT) and titanium tetra-isopropoxide (Ti(O^i^Pr)_4_) precursors have been used to deposit Ti–organic thin films for both battery and biological applications,^4,5,22^ but in combination with few organic precursors only, and mostly aliphatic organics. In general, aromatic organics with more rigid backbones are believed to promote the ideal ALD/MLD surface reactions.^2^ Curcumin – an aromatic diol – was found highly compatible with Ti(O^i^Pr)_4_,^24^ but the relatively high deposition temperature needed is a disadvantage when the target application is based on temperature-sensitive substrates.

In this communication, we report highly promising results for the growth of Ti–organic thin films from Ti(O^i^Pr)_4_ in combination with two different aromatic organic precursors, hydroquinone (HQ) and benzene-1,4-dicarboxylic acid (BDC), see Fig. S1 in ESI.† These hybrid processes have not been explored before for detailed ALD/MLD process parameter optimizations; in the two previous studies involving the precursor combinations, Ti(O^i^Pr)_4_ + HQ and Ti(O^i^Pr)_4_ + BDC, these precursors were utilized for multilayer samples, in the former case to enhance the optical properties of Ti-curcumin films,^24^ and in the latter to enhance the properties of TiO_2_-based electrode coatings.^6^ Here – in addition to the detailed process optimization – we demonstrate the excellent uniformity and chemical stability of the resultant Ti–HQ and Ti–BDC thin films. In particular, we investigate the film growth behaviour using lateral high-aspect-ratio (LHAR)^25–27^ test structures to obtain quantitative data for both the overall penetration depth and the uniformity of film thickness inside the high-aspect-ratio cavities. All these thin-film properties are crucially important for the future sustainable energy applications to be compatible with today's 3D microelectronics technologies. Moreover, we demonstrate the utilization of these ALD/MLD processes for the fabrication of well-defined TiO_2_:organic SL structures from the same sustainable precursors needed for example for flexible barrier layer, photocatalysis and thermoelectric applications.^10,28^

All the depositions were carried out in a flow-type hot-wall ALD reactor (F-120 ASM Microchemistry Ltd); details of the deposition and characterization experiments can be found from the ESI.† Through a systematic approach by mapping the deposition parameters (deposition temperature and precursor pulse lengths) as shown in Fig. 1, both the Ti(O^i^Pr)_4_ + HQ and Ti(O^i^Pr)_4_ + BDC processes could be optimized to yield high-quality Ti–HQ and Ti–BDC thin films, respectively. It should be noted that the heating temperatures needed for the precursor sublimation (*i.e.* 30, 90 and 180 °C for Ti(O^i^Pr)_4_, HQ and BDC, respectively) defined the feasible film deposition temperature ranges; accordingly, the Ti(O^i^Pr)_4_ + HQ process was investigated within the temperature range of 100–200 °C, and the Ti(O^i^Pr)_4_ + BDC process within 190–275 °C, see Fig. 1a. For both processes, a trend typical for most of the ALD/MLD processes was seen,^4,5,15,27^ that is, the growth-per-cycle (GPC) decreased with increasing deposition temperature. For the rest of the basic depositions (excluding the SL studies) we selected the lowest feasible deposition temperatures, *i.e.* 125 °C for Ti(O^i^Pr)_4_ + HQ and 210 °C for Ti(O^i^Pr)_4_ + BDC. Further process optimization with varying precursor pulse lengths (Fig. 1b and c) indicated that the surface saturation condition was achieved with the precursor pulse/purge sequences of 3 s Ti(O^i^Pr)_4_/10 s N_2_/6 s HQ/20 s N_2_ for the Ti–HQ films and 7 s Ti(O^i^Pr)_4_/20 s N_2_/15 s BDC/30 s N_2_ for the Ti–BDC films. Note that we also confirmed that in both cases the GPC values remained unchanged upon increasing the N_2_ purge length (Fig. S2; ESI†).

**Fig. 1:**
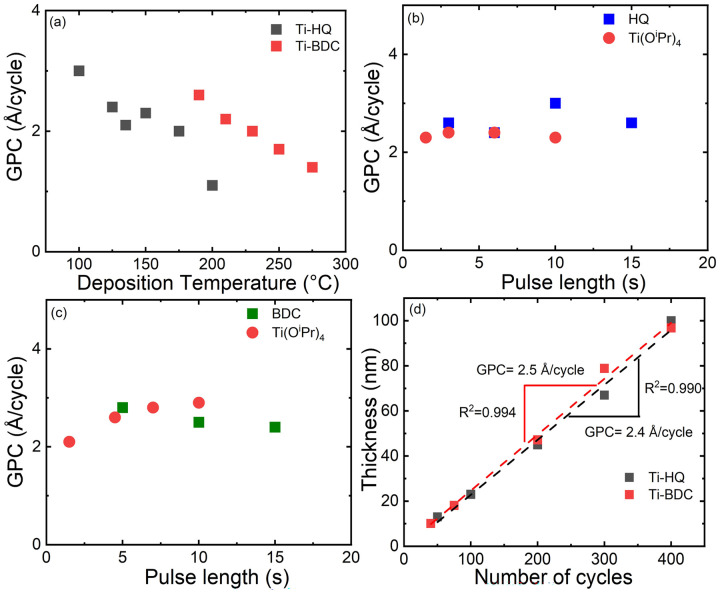
Process characteristics for the two ALD/MLD processes investigated: (a) GPC at various deposition temperatures for both processes, as well as GPC with varied precursor pulse lengths for (b) Ti(O^i^Pr)_4_ + HQ, and (c) Ti(O^i^Pr)_4_ + BDC processes. (d) Demonstration of the linear dependence of film thickness on the number of ALD/MLD cycles applied for both processes.

From Fig. 1d, it is seen that at the chosen temperatures both processes proceed in an essentially ideal manner such that the film thickness increases linearly with increasing number of ALD/MLD cycles applied. From the linear fittings, for the Ti–HQ films the GPC value was determined as 2.4 Å per cycle (at 125 °C) and for the Ti–BDC films as 2.5 Å per cycle (at 210 °C). These GPC values are clearly lower than the “ideal” values expected based on the lengths of the unit-blocks consisting of the Ti atom and the organic unit (9.3 and 10.5 Å for the Ti–HQ and Ti–BDC blocks, respectively), but rather typical for ALD/MLD-grown metal–organic thin films based on benzene-ring backboned organic precursors.^2,3^ Tentatively, we hypothesize that the order of the Ti–organic unit length (9.3 *versus* 10.5 Å) could explain the GPC value order (2.4 *versus* 2.5 Å per cycle) for the Ti–HQ and Ti–BDC films, as the lower deposition temperature (125 *versus* 210 °C) in the former case would have otherwise suggested a higher GPC value for the Ti–HQ films. Another interesting comparison can be made for the Ti–HQ films: the GPC value of 2.4 Å per cycle obtained here for the Ti(O^i^Pr)_4_ + HQ process is lower than the value (4.3 Å per cycle) reported for Ti–HQ films grown with the TiCl_4_ + HQ process at a slightly higher deposition temperature.^19^ We tentatively attribute this to the larger steric hindrance in case of Ti(O^i^Pr)_4_ (due to the larger ligands) as compared to TiCl_4_, and also possibly to their different reactivities the higher reactivity of TiCl_4_ being due to its Lewis acid behaviour and the relatively weak Ti–Cl bond. In future, these speculations could be tackled with DFT-level simulations.

Visually, both the Ti–HQ and Ti–BDC films appeared highly homogeneous, and the expected amorphous nature of the films was confirmed with both GI-XRD (grazing incidence X-ray diffraction) and SEM (scanning electron microscopy) measurements. For the bonding structure analyses, FTIR (Fourier transform infrared) (Fig. 2) and Raman spectroscopy (Fig. S3, ESI†) techniques were employed. For the Ti–HQ films, the absence of the characteristic *ν*(O–H) stretching peak (seen at 3160 cm^−1^ for the HQ precursor)^29^ and the appearance of the *ν*(Ti–O) stretching peak at 499 cm^−1^ confirm that the reaction Ti(O^i^Pr)_4_ + HQ has been complete.^19^ The presence of the *ν*(C=C) stretching vibration at 1486 cm^−1^, the *ν*(C–O) bending vibration at 1199 cm^−1^ and *γ*(C–H) at 833 cm^−1^,^19,29,30^ and the broad *ν*(C–O) peak at 1199 cm^−1^ are all in line with the expected Ti–O–C_6_H_4_–O–Ti bonding sequence in the Ti–HQ films. Similarly, the Raman spectrum shows all the Stokes lines expected for the aromatic ring. Furthermore, the Raman mapping from an area of 270 × 150 μm^2^ affirmed the high film homogeneity as no variation in peak intensities was observed between the data points collected.

**Fig. 2:**
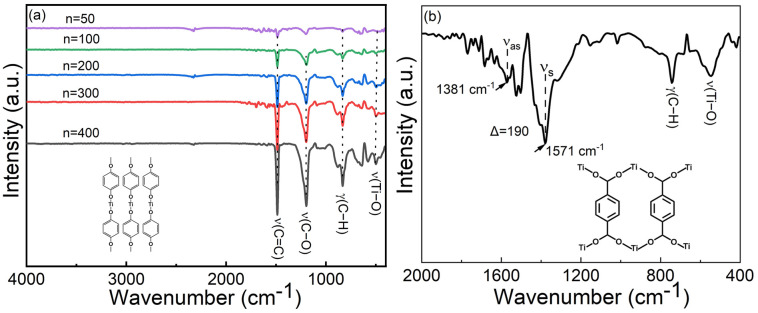
FTIR spectra for (a) Ti–HQ films with different number of ALD/MLD cycles, and (b) a representative Ti–BDC film; the most characteristic vibrations are indicated. Inserts depict the bonding modes between Ti and organics as deducted from the FTIR data; *Δ* (= difference between symmetric and asymmetric carboxylate stretching vibrations) that reflects the type of bonding in case of Ti–BDC is also indicated.

The successful deposition of Ti–BDC films could be verified from the presence of the characteristic FTIR vibrations:^31–33^ C–H out-of-plane bending (741 cm^−1^), ring stretch (1412, 1503 cm^−1^) and Ti–O–C stretching (550 cm^−1^). The symmetric (*ν*_s_) and asymmetric vibration (*ν*_as_) modes of the carboxylate (COO^−^) group were found at 1381 and 1571 cm^−1^, respectively, indicating a bridging-type (*Δ* = 190 cm^−1^) bonding (Fig. 2b inset).^4,33^ All the characteristic peaks corresponding to the aromatic backbone and bridging-type bonding of carboxylate groups could be also confirmed from the Raman spectrum (Fig. S4, ESI†), together with the high film homogeneity from the Raman mapping.

The film density values were determined from XRR data fittings (Fig. S5, ESI†) at 1.3 and 1.9 g cm^−3^ for the Ti–HQ and Ti–BDC films, respectively. Both films were appreciably smooth (roughness <0.2 nm), as typical for amorphous ALD/MLD films.^34^

The film-growth conformality was studied using lateral high-aspect-ratio PillarHall™ test structures with a gap-height (*H*) of 500 nm (Fig. S7, ESI†).^26,27^ For the depositions into these test structures we followed the same pulse/purge parameters as optimized in case of silicon substrates. The film penetration depth (PD) was visualized using both optical microscopy (Fig. S8, ESI†) and SEM (Fig. 3) after peeling off the top-roof membrane using an adhesive tape approach. The contrast difference between the coated and uncoated cavity surface seen in the SEM images was taken as an estimation for the PD value. Moreover, elemental profiles were measured using an energy-dispersive X-ray spectrometry (EDS) through X-ray line scans of the film starting from the opening area and then proceeding further into the cavity (Fig. 3). The PD measured using the contrast SEM indicated that the Ti–BDC film grows deeper inside the cavity (PD: 150 μm) than the Ti–HQ film (PD: 93 μm). These PD numbers correspond to the appreciably high aspect-ratio (PD/H) values of 300 and 186, respectively. For both the films towards the PD ending, a change in contrast in SEM images was observed indicating a reduction in film thickness.^27^ This was further affirmed with the changes in the shape of elemental profiles observed. Elemental profiles (showing the presence of titanium, oxygen and carbon as expected) indicated that the content of the constituting elements decreases along the cavity while remaining constant within the opening area.

**Fig. 3:**
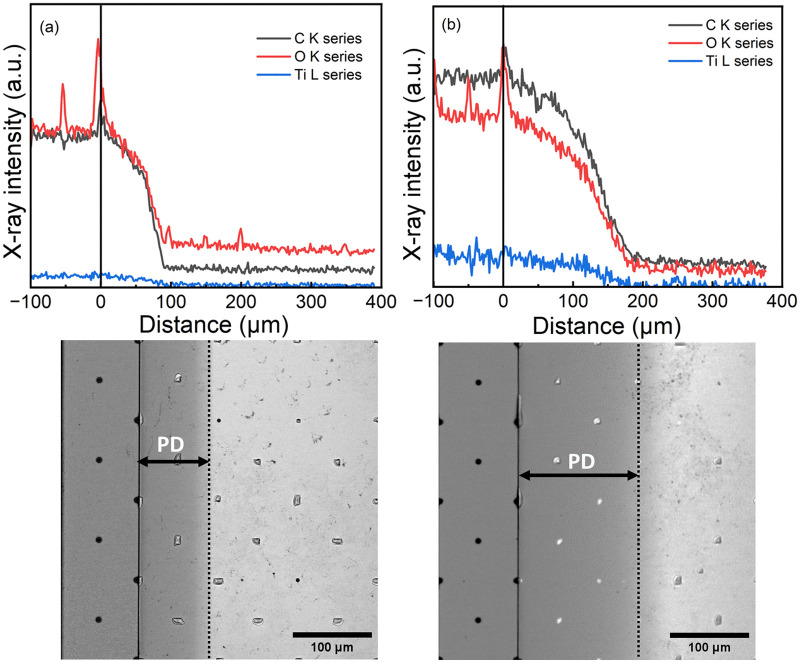
Conformality data: SEM images and corresponding elemental profiles (above) for (a) Ti–HQ and (b) Ti–BDC films. Distance between dots (pillars) is 100 μm. The reduction in film thickness deeper in the trenches is visible with a change in contrast. The observed PD is marked.

We also demonstrated the possibility to combine the Ti(O^i^Pr)_4_ + HQ, and Ti(O^i^Pr)_4_ + BDC processes with ALD TiO_2_ layers grown from the same Ti precursor, *i.e.* Ti(O^i^Pr)_4_ + H_2_O process. For the growth of these TiO_2_:organic SL films, we designed supercycles consisting of 200 cycles of Ti(O^i^Pr)_4_ + H_2_O, followed by a single cycle of Ti(O^i^Pr)_4_ + HQ, or Ti(O^i^Pr)_4_ + BDC. By repeating the supercycle 4 times, SL thin films with 4 organic monomolecular layers embedded within a TiO_2_ matrix were targeted. We carried out the depositions at the lowest feasible temperatures, *i.e.* at 200 and 210 °C for the TiO_2_:HQ, and TiO_2_:BDC films, respectively; these temperatures were defined by the requirements of the Ti(O^i^Pr)_4_ + H_2_O (200 °C) and Ti(O^i^Pr)_4_ + BDC (210 °C) binary processes, respectively. From the XRR patterns (Fig. S9, ESI†) the presence of well-defined SL peaks were evident for both the TiO_2_:HQ and TiO_2_:BDC films. By fitting the XRR data, we determined the GPC values for the TiO_2_ layers, and the thicknesses of the intervening organic layers as follows: GPC(TiO_2_) = 0.26 Å per cycle and HQ-thickness = 7.6 Å for the TiO_2_:HQ SL, and GPC(TiO_2_) = 0.38 Å per cycle and BDC-thickness = 12 Å for the TiO_2_:BDC SL. The observed GPC(TiO_2_) values are within the values reported for the Ti(O^i^Pr)_4_ + H_2_O process under similar temperature window but without organic layers.^35^ Estimations for the organic-layer thicknesses are also in line with previous related cases; 7 Å for HQ in combination with ZnO layers,^36^ and 12 Å for BDC in combination with TiO_2_.^6^ The comparitively thinner HQ layers (in comparison to BCD layers) could be tentatively explained by a tendency of the HQ molecules to bend (rather than stay straight) between the TiO_2_ layers.

Since photocatalysis could be one of future application areas for the TiO_2_:organic films, we carried out UV-vis measurements for our SL films. The absorbance pattern observed (Fig. S10, ESI†) affirmed the influence of organic layers in extending the absorption range to visible region, which is highly beneficial for the intended photocatalytic application.

Finally, we investigated the film stabilities by following the possible changes in the XRR-determined film thickness and FTIR and UV-vis spectral features upon elongated storage in open. For the SL films the FTIR and UV-vis spectra remained essentially unchanged (Fig. S10, ESI†) indicating excellent stability. Also the Ti–BDC films were found appreciably stable (Fig. S6, ESI†) as expected for a metal–carboxylate film.^37^ For the Ti–HQ films, FTIR data (broad *ν*(O–H) band around 3000–3600 cm^−1^) indicated towards water physisorption (Fig. S6, ESI†). We hypothesize that the undercoordinated Ti in Ti–HQ (compared to the bridging-type bonding of BDC to Ti in Ti–BDC) could explain the stronger moisture affinity. However, even after a one-month storage, only a few nm thickness reduction (from 100.3 to 96.5 nm) was observed for the present Ti–HQ films proving them to be clearly more stable than those previously grown from TiCl_4_.^12,13,21^

In conclusion, appreciably stable Ti–organic thin films were obtained through ALD/MLD using the chlorine-free titanium precursor Ti(O^i^Pr)_4_. The film-growth conformality – an essential requirement for applying these coatings in 3D structures – was demonstrated using state-of-the-art lateral high-aspect-ratio test structures and detailed SEM analysis for the overall film penetration depth and elemental mapping. These ALD/MLD processes were moreover shown to be compatible with the Ti(O^i^Pr)_4_ + H_2_O process for TiO_2_, allowing the fabrication of layer-engineered superlattice TiO_2_:organic thin films for future application.

Funding was received from Business Finland (CHEMI-SEMI Co-Innovation project) and the European Union (ERC AdG, UniEn-MLD, No. 101097815). Views and opinions expressed are however those of the authors only and do not necessarily reflect those of the European Union or the European Research Council. Neither the European Union nor the granting authority can be held responsible for them. We thank Chipmetrics for the PillarHall LHAR chips. We acknowledge the continuous use of the RawMatTERS Finland Infrastructure (RAMI), as well as the provided facilities and technical support at OtaNano–Microscopy Center (Aalto-NMC) at Aalto University. T. Jussila and J. Pekkanen are thanked for the XRR measurements.

## Data availability

The data supporting this article have been included as part of the ESI.†

## Conflicts of interest

There are no conflicts of interest to declare.

## Supplementary Material

CC-061-D4CC05425C-s001

## References

[cit1] Gregorczyk K., Knez M. (2016). Prog. Mater. Sci..

[cit2] Multia J., Karppinen M. (2022). Adv. Mater. Interfaces.

[cit3] Meng X. (2017). J. Mater. Chem. A.

[cit4] Vandenbroucke S. S. T., Henderick L., De Taeye L. L., Li J., Jans K., Vereecken P. M., Dendooven J., Detavernier C. (2022). ACS Appl. Mater. Interfaces.

[cit5] Van De Kerckhove K., Mattelaer F., Deduytsche D., Vereecken P. M., Dendooven J., Detavernier C. (2016). Dalton Trans..

[cit6] Ahaliabadeh Z., Miikkulainen V., Mäntymäki M., Colalongo M., Mousavihashemi S., Yao L., Jiang H., Lahtinen J., Kankaanpää T., Kallio T. (2024). Energy Environ. Mater..

[cit7] Li T., Yuan X. Z., Zhang L., Song D., Shi K., Bock C. (2020). Electrochem. Energy Rev..

[cit8] Zhao Y., Zhang L., Liu J., Adair K., Zhao F., Sun Y., Wu T., Bi X., Amine K., Lu J., Sun X. (2021). Chem. Soc. Rev..

[cit9] Ahmed R. A., Carballo K. V., Koirala K. P., Zhao Q., Gao P., Kim J. M., Anderson C. S., Meng X., Wang C., Zhang J. G., Xu W. (2024). Small Struct..

[cit10] Niemelä J. P., Karttunen A. J., Karppinen M. (2015). J. Mater. Chem. C.

[cit11] Heikkinen M., Ghiyasi R., Karppinen M. (2024). Adv. Mater. Interfaces.

[cit12] Kim H., Hyun J., Kim G., Lee E., Min Y. S. (2024). Chem. Mater..

[cit13] Kim H., Hyun J., Min Y. S. (2023). J. Phys. Chem. C.

[cit14] Cao Y. Q., Zhu L., Li X., Cao Z. Y., Wu D., Li A. D. (2015). Dalton Trans..

[cit15] Abdulagatov A. I., Hall R. A., Sutherland J. L., Lee B. H., Cavanagh A. S., George S. M. (2012). Chem. Mater..

[cit16] Yoon K. H., Han K. S., Sung M. M. (2012). Nanoscale Res. Lett..

[cit17] Sood A., Sundberg P., Malm J., Karppinen M. (2011). Appl. Surf. Sci..

[cit18] Nilsen O., Haug K. R., Finstad T., Fjellvåg H. (2013). Chem. Vap. Deposition.

[cit19] Tanskanen A., Sundberg P., Nolan M., Karppinen M. (2021). Thin Solid Films.

[cit20] Sundberg P., Karppinen M. (2014). Eur. J. Inorg. Chem..

[cit21] Lemaire P. C., Oldham C. J., Parsons G. N. (2016). J. Vac. Sci. Technol.
A.

[cit22] Momtazi L., Sønsteby H. H., Dartt D. A., Eidet J. R., Nilsen O. (2017). RSC Adv..

[cit23] Philip A., Ghiyasi R., Karppinen M. (2021). Molecules.

[cit24] Philip A., Ghiyasi R., Karppinen M. (2021). ChemNanoMat.

[cit25] Jain H., Creatore M., Poodt P. (2023). J. Vac. Sci. Technol., A.

[cit26] Madadi M., Heikkinen M., Philip A., Karppinen M. (2024). ACS Appl. Electron. Mater..

[cit27] Philip A., Jussila T., Obenlüneschloß J., Zanders D., Preischel F., Kinnunen J., Devi A., Karppinen M. (2024). Small.

[cit28] Niemelä J. P., Giri A., Hopkins P. E., Karppinen M. (2015). J. Mater. Chem. A.

[cit29] Philip A., Mai L., Ghiyasi R., Devi A., Karppinen M. (2022). Dalton Trans..

[cit30] Niemelä J. P., Karppinen M. (2015). Dalton Trans..

[cit31] Ye G., Sun Y., Zhang D., Zhou W., Lancelot C., Rives A., Lamonier C., Xu W. (2018). Microporous Mesoporous Mater..

[cit32] Ye G., Gu Y., Zhou W., Xu W., Sun Y. (2020). ACS Catal..

[cit33] Tanskanen A., Karppinen M. (2018). Sci. Rep..

[cit34] Klepper K. B., Nilsen O., Fjellvåg H. (2010). Dalton Trans..

[cit35] Ritala M., Leskelä M., Niinistö L., Haussalo P. (1993). Chem. Mater..

[cit36] Ghiyasi R., Milich M., Tomko J., Hopkins P. E., Karppinen M. (2021). Appl. Phys. Lett..

[cit37] Ahvenniemi E., Karppinen M. (2016). Dalton Trans..

