# Supplementary material for: Highly stable and conformal Ti–organic thin films from sustainable precursors via atomic/molecular layer deposition towards green energy applications
Source: Chem Commun (Camb). 2024 Dec 6;61(5):917–20. doi: 10.1039/d4cc05425c (PMC11638904; doi:10.1039/d4cc05425c)
Supplement: CC-061-D4CC05425C-s001 [file CC-061-D4CC05425C-s001.pdf]

Supplementary Information (SI) for ChemComm.
This journal is © The Royal Society of Chemistry 2024

Electronic Supplementary Information (ESI)

Highly stable and conformal Ti-organic thin films from sustainable precursors via atomic/molecular layer deposition towards green energy applications

Anish Philip, Umaid Lone, Olga Partanen, Eero Haimi, and Maarit Karppinen*

[a] *Department of Chemistry and Materials Science, Aalto University, P.O. Box 16100, FI-00076 Espoo, Finland. E-mail: maarit.karppinen@aalto.fi*

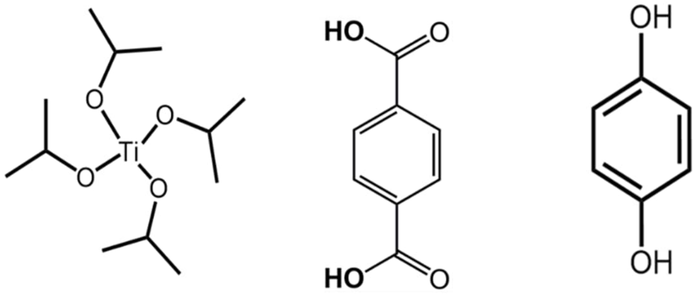

$Ti(O^iPr)_4$ **BDC** **HQ**

Figure S1. Molecular structures of the precursors: titanium isopropoxide ($Ti(O^iPr)_4$), benzene-1,4-dicarboxylic acid (BDC) and hydroquinone (HQ).

Experimental Procedure

All the thin films were deposited on 2.0 × 2.0 cm^2 silicon substrates in a commercial flow-type hot-wall ALD reactor (F-120 by ASM Ltd). Those SL films studied for absorbance were also deposited on borosilicate glasses. In these ALD/MLD processes, the following precursors were used: titanium (IV) isopropoxide ($Ti(O^iPr)_4$, Aldrich, 97%), deionized water, hydroquinone (HQ; Sigma-Aldrich, ≥99.5 %), and benzene-1, 4-dicarboxylic acid (BDC; Tokyo Chemical Industry CO., Ltd., >99.0 %). $Ti(O^iPr)_4$,HQ and BDC were placed inside the reactor in open boats and heated at 30, 90, and 180 °C, respectively, while the deionized water cylinder was placed outside the reactor and pulsed at RT. Nitrogen (N_2, 99.999%, flow rate at 300 SCCM, Schmidlin UHPN 3000 N_2 generator) was used as both the carrier and purge gas for the deposition process. The reactor pressure was kept at 3-6 mbar.

Superlattice (SL) structures were deposited using supercycles consisting of both ALD ($Ti(O^iPr)_4$ +H_2O) cycles for TiO_2 layers and MLD ($Ti(O^iPr)_4$ +HQ or BDC) cycles for the molecular organic layers. For the ALD TiO_2 layers, the pulse and purge lengths were adopted from our previous work.[1] For Ti-HQ molecular layers the precursor pulse/purge sequence was 3 s $Ti(O^iPr)_4$ / 10 s N_2 / 6 s HQ / 20 s N_2, and for the Ti-BDC molecular layers the pulse/purge sequence was 7 s Ti(OiPr)4/ 20 s N_2 / 15 s BDC / 30 s N_2. The pulsing sequence followed the pattern: $[(Ti(O^iPr)_4 + H_2O)_m + (Ti(O^iPr)_4 + organic)]_n + (Ti(O^iPr)_4 + H_2O)_m$. Here m controls the thickness of individual TiO_2 layers in the superlattice and n controls the total number of organic layers within the TiO_2 matrix. In the current communication, the opted m and n were 200 and 4 respectively. Deposition temperature was 200 °C for the TiO_2:HQ SL films, and 210 °C for the TiO_2:BDC SL films.

Characterization Techniques

The thickness of the Ti-organic hybrid films was determined with X-ray reflectivity (XRR; PANalytical X'Pert PRO Alfa 1); the data were fitted with the X'Pert Reflectivity software by PANalytical. The growth-per-cycle (GPC) value was obtained by dividing the overall film thickness with the number of deposition cycles. The same device was used to collect the grazing incidence X-ray diffraction (GIXRD) patterns with an incident angle of 0.5°. For the verification of the superlattice (SL) pattern we used Rigaku Smartlab X-ray diffractometer.

In order to verify the presence of organic species and investigate the bonding types between the organic and inorganic components both Fourier transform infrared (FTIR, Bruker alpha II) spectroscopy and Raman (Witec Raman with a 532 nm excitation wavelength) were used. The FTIR measurements were performed in a transmission mode in the range of 400–4000 cm^{-1}. The resolution was 4 cm^{-1} and each given spectrum was an average of 32 measured spectra. The interference from the silicon substrate were removed by subtracting the FTIR spectrum of the bare silicon substrate from the FTIR spectra of the samples. Shimadzu UV-2600 spectrometer was used for recording the UV–vis absorbance spectra (wavelength range of 200–800 nm) for the SL films deposited on borosilicate glass plates.

Scanning electron microscope (SEM, Tescan Mira3) was used for analyzing the surface morphology of the films. For the SEM measurement the sample specimen was mounted on a carbon tape and sputtered an Au–Pd mixture on top of it. The analysis was performed at an acceleration voltage of 10 kV.

For investigating the conformality characteristics, depositions were performed on PillarHall® LHAR (LHAR5) test structures (Chipmetrics Ltd).[2,3] A reference silicon piece was also placed together with a LHAR substrate in the reactor, and the film thickness on this reference substrate was determined using XRR. The advantage of the LHAR chips is the easier film growth investigation as these chips do not require cross sectioning, which is unavoidable with the conventional vertical-hole approach. In the case PillarHall LHAR chips, an adhesion tape was used for removing the top-roof polysilicon membrane from the LHAR structure after the thin-film deposition. For this procedure, a small piece of adhesion tape was gently glued on chip and then pulled it away to peel off the polysilicon membrane and thereby exposing the film for further analysis. Optical microscopy (Euromex Holland) was used for imaging the film grown on the LHAR substrate and to get an estimate on the penetration depth. For further analysis of penetration depth (PD) and conformality, combined SEM and energy-dispersive X-ray spectrometry (EDS) examination was carried out using JEOL JSM-IT800HL scanning electron microscope fitted with Oxford Instruments X-ray spectrometer. No sample preparation for charging prevention was needed. In the examination, backscattered electron imaging of sample surfaces was carried out first. Subsequently, X-ray line scans of elemental profiles were measured. Presented X-ray line scans intensities are directional averages of areal measurements.

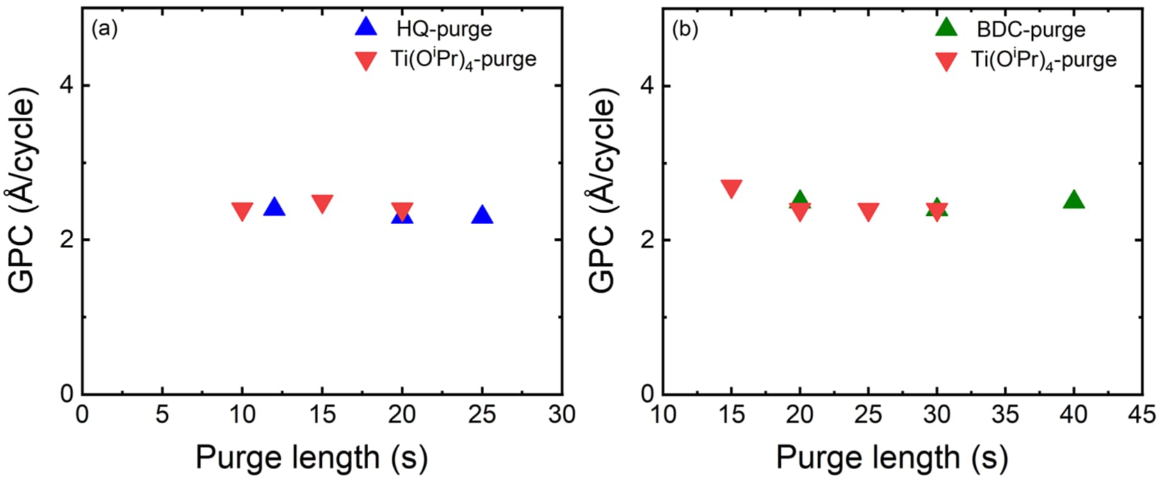

Figure S2. GPC versus purge lengths after precursor pulses for (a) Ti(O^{i}Pr)$_4$+ HQ and (b) Ti(O^{i}Pr)$_4$+ BDC process.

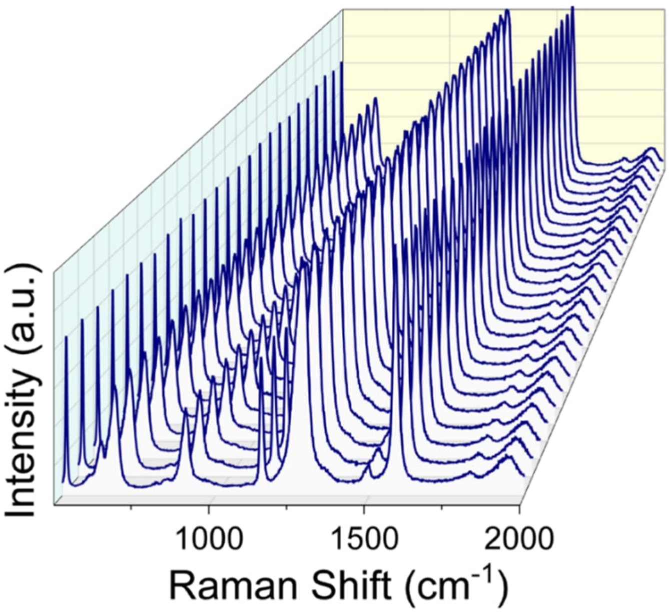

Figure S3. Mapping of Raman spectra for 25 data points collected from an area of 270 µm x 150 µm for a representative Ti-HQ thin film.

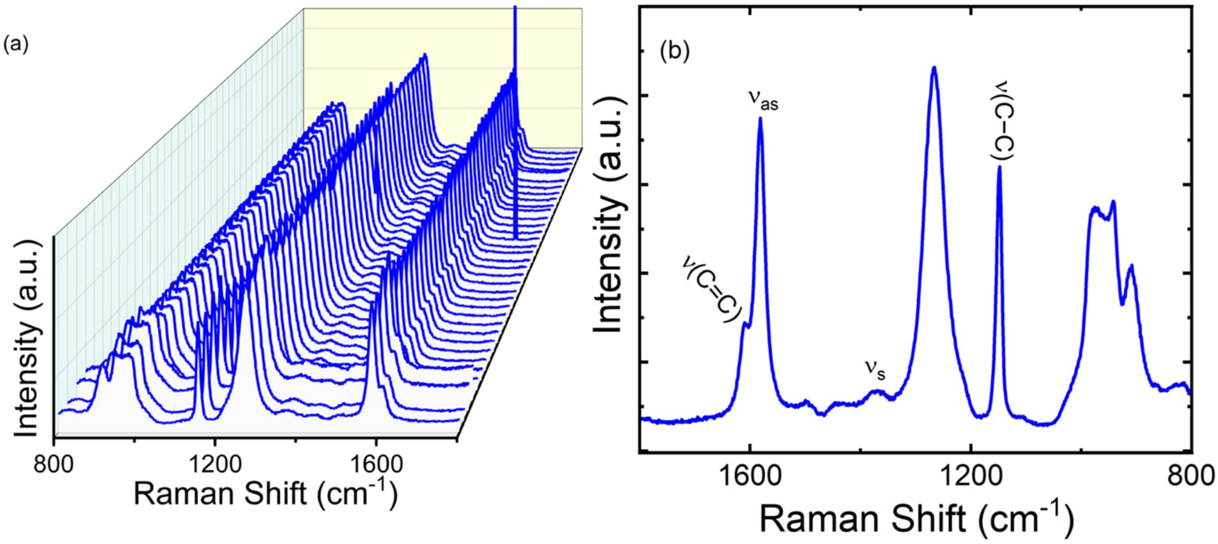

Figure S4. Raman analysis of Ti-BDC film: (a) Mapping of Raman spectra for 25 data points collected from an area of 100 µm x 75 µm for a representative Ti-BDC thin film. Characteristic Raman vibrations affirming the expected Ti-BDC structure are marked.

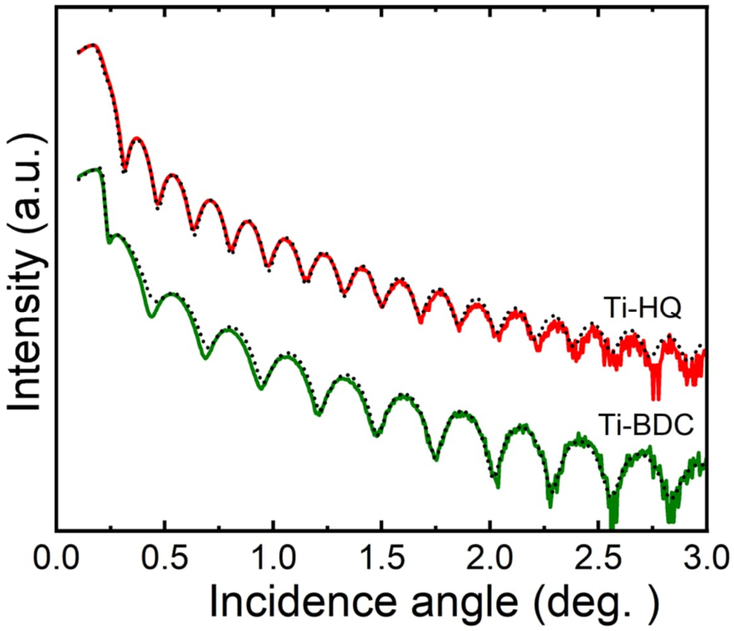

Figure S5. Comparison of fitted and experimental XRR patterns for representative Ti-HQ and Ti-BDC thin films; the dotted lines are fitted patterns.

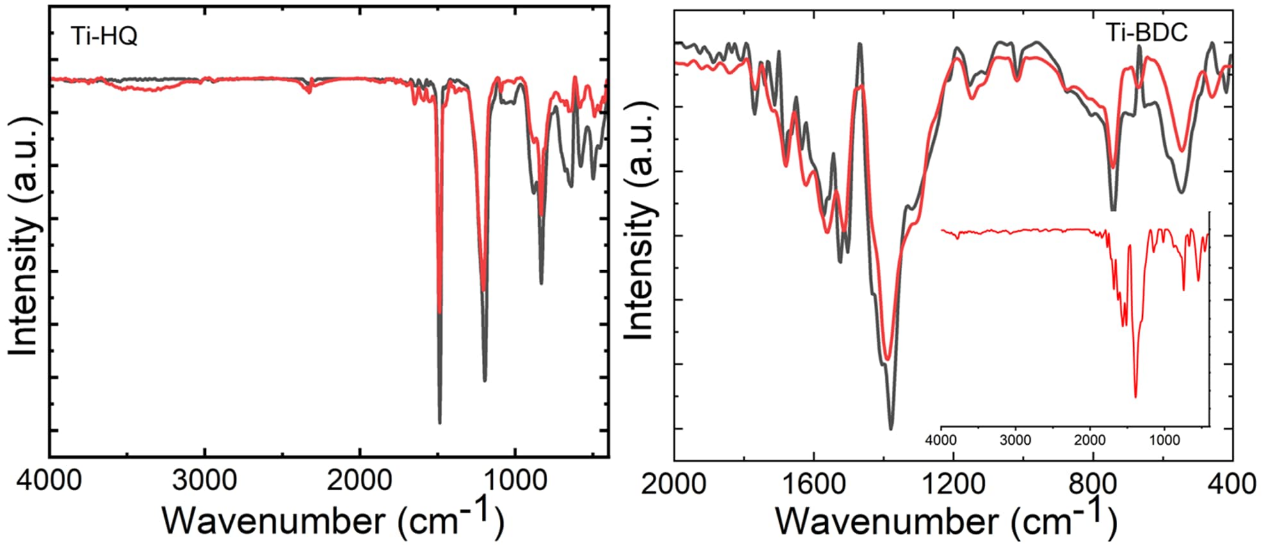

Figure S6. Comparison of FTIR spectrum from as deposited (black lines) and aged (red lines) for both Ti-HQ films(left) and Ti-BDC film (right). FTIR spectrum for the whole wavelength region studied in case of aged Ti-BDC film given as inset.

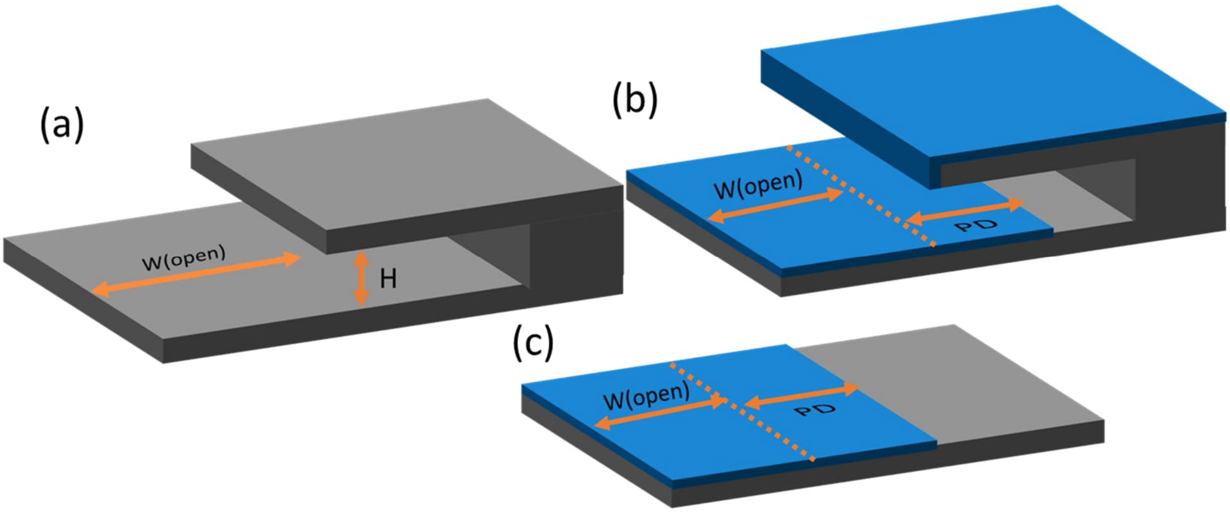

Figure S7. Schematic illustration of top-view of conformality analysis using PillarHall LHAR test structures: (a) Test structure before ALD/MLD coating, (b) after ALD/MLD coating, and (c) after ALD/MLD coating and peeling off the roof membrane; W(open) stands for the opening area width, H for gap height (500 nm) and PD for penetration depth. The dashed lines in (b) and (c) correspond to the membrane edge which separates the cavity from the opening area.

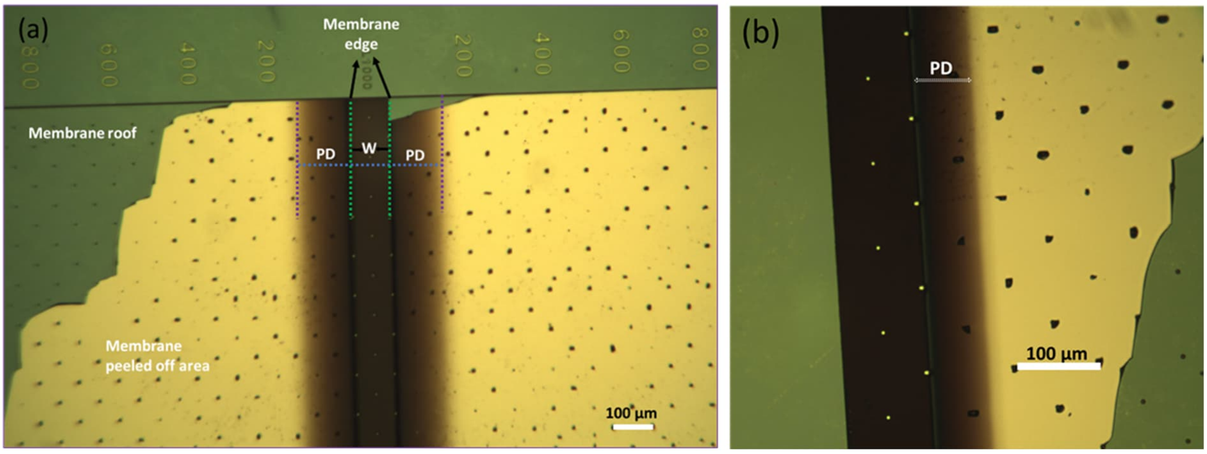

Figure S8. Optical microscopy images indicating the penetration depth (contrast difference between the coated and non-coated surfaces: (a) Ti-BDC film deposied in a mirrored cavity and (b) Ti-HQ deposied in a non mirrored cavity. The opening area (W), membrane edge (where the cavity begins), penetration depth (PD, the extent to which the film grows inside the cavity, membrane roof and membarne peeled off area are marked. The distance between the pillars is 100 µm.

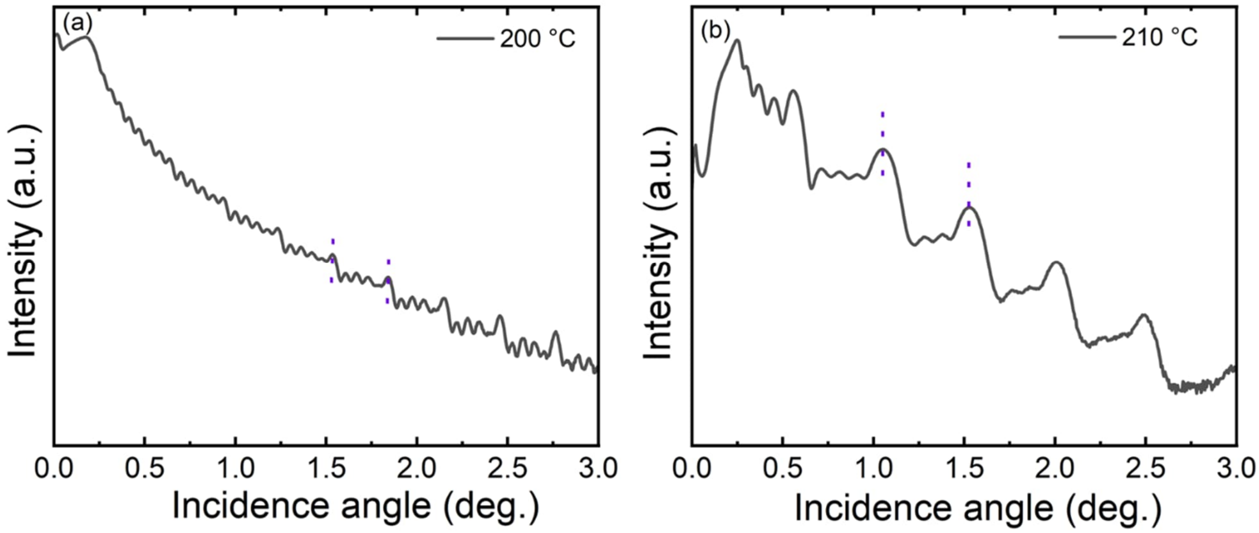

Figure S9. XRR pattern from (a) TiO_2:HQ (deposited at 200 ºC), and (b) TiO_2:BDC (deposited at 210 ºC) superlattice thin films. The SL peaks are indictaed with the dashed lines.

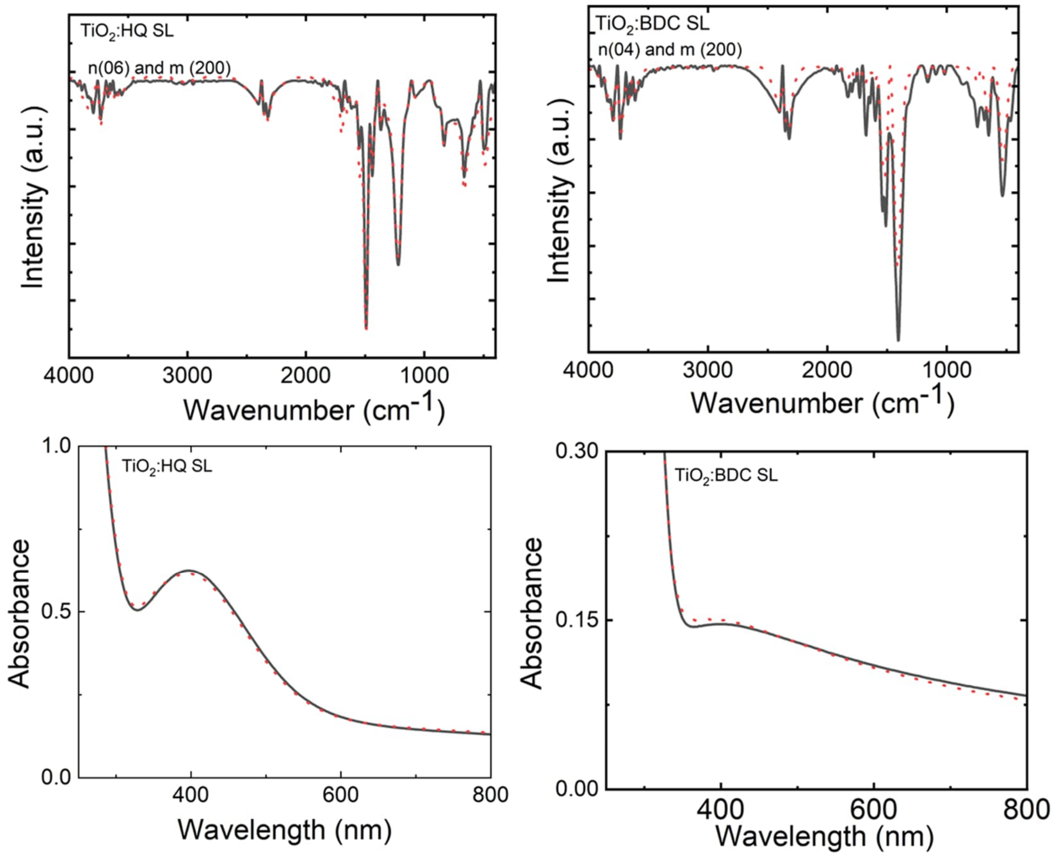

Figure S10. Comparison of both FTIR (top row) and UV-vis (bottom row) data from as deposited (black lines) and aged (dotted red lines) TiO_2:HQ and TiO_2:BDC SL films.

Notes and references

1 A. Philip, R. Ghiyasi and M. Karppinen, *ChemNanoMat*, 2021, 7, 253–256.

2 F. Gao, S. Arpiainen and R. L. Puurunen, *J. Vac. Sci. Technol. A,* 2015, 33, 010601.

3 H. Jain, M. Creatore, P. Poodt, *J. Vac. Sci. Technol. A* 2023, *41*, 012401
